# Comparison of web-based and face-to-face interviews for application to an anesthesiology training program: a pilot study

**DOI:** 10.5116/ijme.56e5.491a

**Published:** 2016-04-03

**Authors:** Marissa G. Vadi, Mathew R. Malkin, John Lenart, Gary R. Stier, Jason W. Gatling, Richard L. Applegate II

**Affiliations:** 1 Department of Anesthesiology, Loma Linda University School of Medicine, Loma Linda, California, 92354, USA

**Keywords:** Graduate medical education, interviews, recruitment, residency, videoconferencing

## Abstract

**Objectives:**

This study compared admission rates to a United States anesthesiology residency program for applicants completing face-to-face versus web-based interviews during the admissions process. We also explored factors driving applicants to select each interview type.

**Methods:**

The 211 applicants invited to interview for admission to our anesthesiology residency program during the 2014-2015 application cycle were participants in this pilot observational study. Of these, 141 applicants selected face-to-face interviews, 53 applicants selected web-based interviews, and 17 applicants declined to interview. Data regarding applicants' reasons for selecting a particular interview type were gathered using an anonymous online survey after interview completion. Residency program admission rates and survey answers were compared between applicants completing face-to-face versus web-based interviews.

**Results:**

One hundred twenty-seven (75.1%) applicants completed face-to-face and 42 (24.9%) completed web-based interviews. The admission rate to our residency program was not significantly different between applicants completing face-to-face versus web-based interviews. One hundred eleven applicants completed post-interview surveys. The most common reasons for selecting web-based interviews were conflict of interview dates between programs, travel concerns, or financial limitations. Applicants selected face-to-face interviews due to a desire to interact with current residents, or geographic proximity to the residency program.

**Conclusions:**

These results suggest that completion of web-based interviews is a viable alternative to completion of face-to-face interviews, and that choice of interview type does not affect the rate of applicant admission to the residency program. Web-based interviews may be of particular interest to applicants applying to a large number of programs, or with financial limitations.

## Introduction

Medical education extends beyond completion of a medical degree in many countries.  To become a practicing physician and to specialize within a distinct medical discipline, additional post-graduate clinical training must be completed.  This training is known as "residency" in the United States (US) and Canada, while in the United Kingdom, physicians-in-training complete two years of work-based education in the Foundation Programme before proceeding to specialty / general practitioner training.  In each country, applicants to post-graduate medical training programs often complete both written applications and face-to-face interviews at prospective training program sites as part of the admissions process.^1^^-^^3^ Applications to US residency programs are coordinated through the National Resident Matching Program (NRMP).^1^ Medical students begin the application process during the final year of medical school by submitting standardized written applications to a central application service. After reviewing applications for academic merit, residency programs invite select candidates for face-to-face interviews. 

At the conclusion of the interview period, residency applicants submit a "rank-order list" to the NRMP.  The rank-order list indicates a list of programs where the applicant wishes to train ranked in his/her order of preference.  Each residency program also submits a rank-order list indicating a list of applicants the program wishes to train ranked in the program's order of preference. The process is blinded so that neither applicant nor residency program will see the other's list.  A computer algorithm then assigns each applicant to a residency position using data obtained from the rank-order lists.  The NRMP application process closely mirrors that of the Canadian Resident Matching Service used by Canadian residency programs.^2^

Anesthesiology is an increasingly competitive medical specialty in the US. As such, 49 senior US medical school students failed to secure a desired residency position in anesthesiology through the NRMP in 2014.^4^ The probability of admission to a residency program in anesthesiology increases with the number of contiguous program ranks made by a specific applicant: US medical school seniors admitted to a post-graduate residency program in anesthesiology through the NRMP in 2014 ranked an average of 14.4 programs on their rank-order lists.^4^ It is clear that applicants to anesthesiology residency positions often apply to multiple training programs to remain competitive in the admissions process.

Traditionally, applying to US anesthesiology residency programs has required applicants to participate in face-to-face interviews with faculty members at each prospective residency program.  Face-to-face interview attendance poses several disadvantages for residency applicants.  Time away from medical studies decreases educational productivity. The residency interview season is abbreviated, and some applicants experience scheduling conflicts between interview dates at different residency programs. Traveling to multiple out-of-town interviews may result in financial hardship.^5^^,^^6^ Candidates may be unable to accept interview offers they otherwise would have accepted due to these constraints, thus failing to capitalize on important opportunities to market themselves to residency programs.^7^    

Web-based residency interviews have been proposed as an alternative to traditional face-to-face interviews to maximize applicants’ interview opportunities. Videoconferencing software applications have been used to conduct residency interviews in other medical specialties, generally in conjunction with a face-to-face interview on the same day or on a day shortly after the web-based interview.^5^^,^^8^^-^^10^ The impact of web-based interviews on residency program admission results is unclear. A 2014 NRMP survey of US anesthesiology residency program directors cited interactions with faculty during interview and visit, interpersonal skills, and interactions with current residents during interview and visit as the three top factors determining an applicant's position on the residency program rank-order list.^11^ Some residency applicants may hesitate to select web-based residency interviews due to a belief that not participating during face-to-face residency interview events will negatively impact the chance of admission to their preferred residency program.^12^ Prior investigations of web-based interviews focused on cost-effectiveness and post-interview applicant satisfaction, but did not provide residency program admission data.  This pilot study was designed to compare admission rates to a US anesthesiology residency program for applicants completing face-to-face versus web-based interviews as part of the residency admissions process.   

## Methods

### Study design

We performed a prospective observational study comparing NRMP admission rates to the Loma Linda University anesthesiology residency program between applicants completing face-to-face versus web-based admission interviews.  Ethical approval to complete the study was obtained from the Loma Linda University Institutional Review Board.

### Study participants

**Figure 1:**
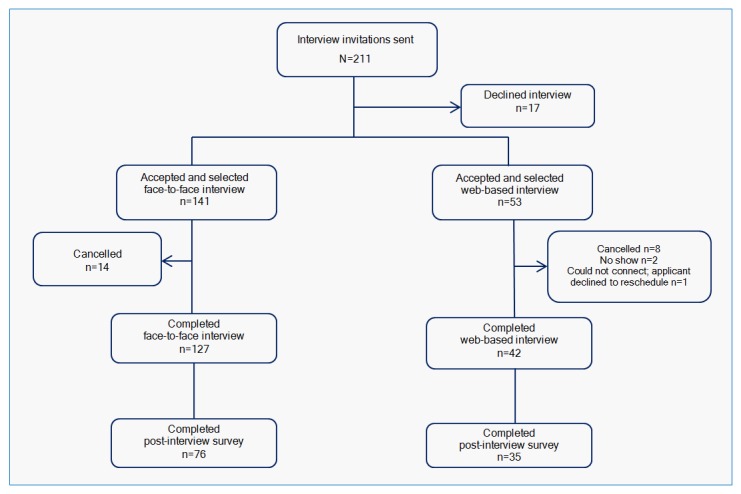
Distribution of study participants

The 211 applicants invited to interview for admission to the Loma Linda University anesthesiology residency program during the 2014-2015 NRMP application cycle were eligible for study participation.  Applicants chose to complete either a face-to-face or a web-based residency selection interview.  Of the 211 applicants invited to interview, 141 (72.7%) selected face-to-face and 53 (27.7%) selected web-based interviews.  Fourteen (9.9%) face-to-face and 11 (20.8%) web-based interview applicants did not complete interviews. Thus, 127 (75.1%) applicants completed face-to-face and 42 (24.9%) completed web-based interviews.  The 169 applicants who completed either face-to-face or web-based interviews were invited to complete post-interview surveys regarding their interview experiences.  This is illustrated in Figure 1.

### Data collection

Applicant demographic data such as age, gender, United States Medical School Licensing Examination Step 1 score, and medical school location were obtained from written application forms completed by all applicants to the residency program. The residency program coordinator maintained a database indicating the type of interview (face-to-face versus web-based) selected and completed by each applicant.  A list of admitted applicants was provided to the residency program by the NRMP after applicant and residency program rank-order lists were analyzed by the NRMP computer algorithm.

A brief Internet survey was developed by the authors and aimed to explore applicants' reasons for choosing either face-to-face or web-based interviews. An invitation to complete this survey was provided to all applicants who completed residency admission interviews after interview completion. Survey participation was voluntary and anonymous. Applicants were informed in writing that participation would not affect the residency application process. 

### Procedures

Interview offers were extended to residency applicants via standardized electronic mail that included a list of available interview dates, each designated as a face-to-face interview or a web-based interview day. Applicants selected their preferred interview dates.  The interview invitation included a statement advising residency applicants that face-to-face and web-based interviews would be considered equivalent by the faculty members determining the residency program rank-order list.

### Face-to-face interviews

Applicants who selected face-to-face interviews were invited to attend an optional off-campus dinner with current anesthesiology residents the night before their interviews. On the day of the interviews, the residency program director provided a comprehensive program overview via PowerPoint slide presentation. Applicants completed three or four 10-minute interviews with faculty members. These non-structured interviews were not recorded to protect applicant privacy, but faculty made notes and provided summary evaluations of their judgments of applicant suitability for admission to our residency program that were later considered when the final program rank-order list was formulated. The face-to-face applicants toured the medical center, and participated in a lunchtime question and answer session with current residents. 

### Web-based interviews

Applicants who selected web-based interviews were provided access to audio and video versions of the comprehensive program overview given to face-to-face interview applicants by the residency program director.  They were also provided access to a video tour of Loma Linda University Medical Center and its surrounding communities.

Web-based interview applicants selected either FaceTime or Skype as their preferred videoconferencing application. Applicants were allowed their choice of electronic device and Internet connection. They were allowed to use headphones and/or external microphones as needed to improve audio quality. All Department of Anesthesiology videoconferencing connections were established using desktop iMac computers located in separate rooms and hard-wired to the high-speed university Ethernet network. Like face-to-face applicants, web-based interview candidates completed three or four 10-minute interviews with faculty members.  The same six faculty members who conducted face-to-face interviews conducted web-based interviews.  The non-structured web-based interviews were not recorded to protect applicant privacy, but faculty made notes and provided summary evaluations of their judgments of applicant suitability for admission to our residency program that were later considered when the final program rank-order list was formulated. Web-based interview applicants were also invited to interact with current anesthesiology residents through audio-visual Google Hangouts chat sessions conducted twice on each web-based interview day.

All applicants (face-to-face and web-based interview) were advised they could schedule an optional on-campus department tour at their convenience on a day subsequent to their interview. However, such visits were not required for applicants to be placed on the program rank-order list.

### Post-interview survey

Following interview days, all applicants received e-mail invitations to participate in an anonymous web-based survey focusing on the applicants' reasons for selecting a face-to-face or a web-based interview and their perceptions of the efficacy of each interview type.  No identifying data were collected and results were not analyzed until after NRMP admission results were announced.

### Residency program rank-order list formation

The residency program rank-order list was compiled by the six faculty interviewers, none of whom interviewed all applicants, and the department chairman. Factors considered included, but were not limited to, faculty interviewer summary comments, United States Medical Licensing Exam Step 1 scores, medical school evaluations, and letters of recommendation.  The faculty members who were involved in rank-order list formation were given summaries of interviewer impressions and comments that did not include information regarding whether the candidates completed face-to-face or web-based interviews. The finalized rank-order list was then submitted to the NRMP.

### Data analysis

The proportion of applicants who were admitted to our anesthesiology residency program through the NRMP was compared between those who completed face-to-face and those who completed web-based interviews. Differences in answers to survey questions were compared between applicants who completed face-to-face and those who completed web-based interviews. Continuous data were analyzed for normality using the Shapiro-Wilk test and expressed as either mean [95% Confidence Interval (CI)] or median [95% CI]. Normally distributed data were analyzed using the t-test. Data that were not normally distributed were analyzed by the Wilcoxon rank-sum test and are expressed as the smoothed empirical likelihood median [95% CI]; the Hodges Lehman method, assuming data symmetry, was used to compare differences. Categorical data were analyzed by Chi-square test, and differences were compared using the Wald Test for two proportions. Statistical significance was taken at p <0.05 (JMP 10.0.0, SAS Institute, Cary, NC, USA).

## Results

There were no between group differences in applicant characteristics including United States Medical Licensing Examination Step 1 scores (Table 1). A larger proportion of face-to-face applicants attended a medical school in California, the same state as our residency program (p=0. 000016). However, the overall regional distribution of medical schools was not different from the prior 4 years of applicants to our program. A larger proportion of web-based applicants completed a post-interview campus visit (p=0.0025).

**Table 1:** Residency program applicant characteristics, N=169. Loma Linda University, Loma Linda, California, United States, 2014-2015

Applicant characteristics	Face-to-face interview (n = 127)	Web-based interview (n = 42)	p-value
Gender			
Female, n (%)	39 (30.7%)	17 (40.5%)	0.244
Male, n (%)	88 (69.3%)	25 (59.5%)	
Age in years, Median, 95% CI^*^	27.1, 26.7 to 27.8	27.2, 26.6 to 28.4	0.746
USMLE^**^ Step 1 score, Mean, 95% CI	232.0, 229.7 to 234.3	229.1, 225.2 to 233.1	0.229
Medical school located in California, n (%)	47 (37.0%)	1 (2.4%)	0. 000016
Completed post-interview campus visit, n (%)	3 (2.4%)	7 (16.7%)	0.0025
Assigned to upper half of residency program rank-order list, n (%)	61 (48.0%)	23 (54.8%)	0.450
Admitted to residency program through NRMP^†^, n (%)	14 (11.0%)	3 (7.1%)	0.568

*CI: Confidence Interval; **USMLE: United States Medical Licensing Examination; †NRMP: National Resident Matching Program.

Choice of web-based versus face-to-face interview was not associated with a difference in the proportion of applicants admitted to our residency program through the NRMP (Table 1). An equal proportion of applicants were assigned to the upper half of the program rank-order list, regardless of the type of interview completed. Only one of 3 web-based and one of 14 face-to-face interview candidates who were ultimately admitted to our residency program completed a post-interview visit.

Post-interview surveys were completed by 111 applicants (76 face-to-face and 35 web-based interview applicants).  One hundred three applicants provided answers to questions exploring factors driving applicants to select each interview type. The most common self-reported reasons for selecting web-based interviews were conflict of interview dates between programs, geographic/travel concerns, or financial limitations (Table 2 Applicants largely selected face-to-face interviews due to a desire to interact with current residents, geographic proximity, or a desire to visit the campus/facility.

Of web-based interview survey respondents, 3 (9.4%) reported difficulty in maintaining eye contact; 2 (6.3%) reported sub-optimal video quality; and 1 (3.1%) reported sub-optimal audio quality during their interview. Still, web-based interviews met or exceeded expectations of all submitting survey responses. Similarly, all but 2 face-to-face interview applicants who completed surveys felt their interview experience was good or excellent.

**Table 2:** Residency applicants' self-reported reasons for selecting face-to-face versus web-based interviews, N=103. Loma Linda University, Loma Linda, California, United States, 2014-2015

Completed face-to-face interview (n=71)
Reasons for selecting face-to-face interview	1. Want to interact with current residents (45.1%)
	2. Geographic proximity (29.6%)
	3. Want to see campus / facility (11.3%)
	4. Want to evaluate surrounding neighbourhood (7.0%)
	5. Fear web-based interview will negatively impact chance of admission (4.2%)
	6. Other (2.8%)
Completed web-based interview (n=32)
Reasons for selecting web-based interview	1. Conflict of interview dates (31.3%)
	2. Geographic / travel concerns (28.1%)
	3. Financial limitations (25.0%)
	4. Unable to get time off (9.4%)
	5. Enjoy web-based communication (3.2%)
	6. Other (3.0%)

## Discussion

Residency admission interviews are viewed as important factors influencing rank-order list formation by both residency programs^13^^-^^20^ and applicants.^21^^,^^22^ Residency programs use interviews to assess applicants’ noncognitive skills, such as interpersonal and communication skills, maturity, interest in the field, and honesty.^13^^,^^23^ Applicants value interactions with current residents as important opportunities to assess resident morale and program weaknesses as perceived by current trainees.^24^^,^^25^ However, these perceptions that resident interviews are key components of the residency selection process are not always supported by the available literature.  In three studies, interviews changed the rank-order list position of applicants more than 10 positions below or above their preinterview rank.^26^^-^^28^ However, one study found such a strong correlation between the interview and academic variables that final rank was unchanged whether the interview was added to the statistical model or not.^29^ Interviewers not blinded to applicants’ academic records may subconsciously base their interview assessments on academic criteria. Interview performance is also not predictive of an applicant’s subsequent clinical performance, as outlined in a review of 34 studies on residency interviews.^30^ This review, noting the high financial cost and poor predictive power of residency interviews, cited use of web-based residency interviews as an area of future study in hopes of diminishing interview expenses for both residency programs and applicants. 

In our pilot study, we found that completing a web-based versus a face-to-face residency selection interview did not affect the proportion of applicants who were accepted to our anesthesiology residency program through the NRMP.  Web-based interview and face-to-face interview applicants were equally likely to be ranked in the upper or lower half of the program rank-order list. Internal residency program data from the prior 3 years shows that applicants who gained admission to our program in those years were ranked in the upper half of the program rank-order list. Scheduling, geographic/travel, and financial issues were the most common self-reported considerations leading applicants to select web-based interviews. All web-based interview applicants who completed anonymous post-interview surveys perceived web-based interviews to be effective, with a majority indicating a preference for this interview type over face-to-face interviews. While videoconferencing software applications have been used to conduct residency interviews for other medical specialties, our study addresses several questions left unanswered by prior investigations.

First, we address the use of web-based interviews for all candidates considered qualified for admission to our residency program. Ours is a larger cohort of applicants (169 completed interviews) than prior reports that only included up to 48 applicants.^9^ We offered the opportunity to choose face-to-face or web-based interviews to all applicants invited to interview, rather than using web-based interviews as a screening tool for applicants felt to be less qualified for admission to the residency program as previously reported.^5^

Second, our study investigated the impact of an applicant completing either a web-based or a face-to-face interview, rather than both types of interviews. We did not require that applicants completing web-based interviews schedule a post-interview campus visit as in prior comparisons of web-based and face-to-face interviews.^5^^,^^10^

Third, we report admission rates to our residency through the NRMP. Prior studies of 33 urology residency applicants who completed both face-to-face and web-based interviews^10^ and of 16 gastroenterology fellowship applicants who also completed both face-to-face and web-based interviews^8^ focused on applicant satisfaction but did not report admission rates. A study of 48 ophthalmology residency applicants allowed self-selection of face-to-face versus web-based interview.^9^ In that study, 21 (44%) chose a web-based interview and 12 (57%) of those also completed a post-interview campus visit. While there was no significant difference in the number of web-based interview versus face-to-face interview applicants ranked in the top 25 spots on the program rank-order list, final admission data were not presented.  The residency program rank-order list is important to show that the program is not biased, but does not indicate whether web-based candidates will rank a program highly on their rank-order lists. Residency program directors may perceive that web-based interview applicants either lack serious interest in the residency program or could fail to rank the residency program highly because they did not develop interpersonal connections with the program faculty or residents during an in-person visit. The equivalent admission rate between candidates completing web-based and face-to-face interviews suggests that web-based interview applicants were committed candidates who ranked our program highly enough to gain admission.     

Several factors limit generalizing the findings of our study. This was a single-center investigation using one year of NRMP applications and admission results, in which applicants were not randomized to complete a particular interview type. While this allowed us to better characterize the type of applicant desiring web-based interviews, it is unclear whether residency applicants' perceptions of interview efficacy would change if they were not assigned to their first choice of interview type. The characteristics of applicants to our anesthesiology residency program may differ from those applying to other anesthesiology residency programs, limiting external validity. Importantly however, we interviewed candidates from a wide range of medical schools across the United States, and those who completed surveys self-reported application to nearly one-third of United States anesthesiology residency programs. Further, the average United States Medical Licensing Examination Step 1 scores of our applicants (Table 1) were similar to the average score (230) reported from all applicants who were admitted to US anesthesiology programs in 2014.^1^ This suggests that the applicants studied here are similar to those applying to other anesthesiology residency programs in the US. Randomized, multi-center studies are needed to determine more definitively whether web-based interviews are a viable alternative to traditional face-to-face interviews for all, or only for a subset of applicants and residency programs.

## Conclusions

Completing a web-based versus a face-to-face residency selection interview did not affect the admission rate to our anesthesiology residency program.  Applicants who chose web-based interviews indicated that scheduling, travel or financial concerns were important considerations motivating this choice. Taken together, these findings suggest that for at least some applicants, offering web-based interviews could provide applicant benefit without harming chances of acceptance to a residency program.  Future studies should focus on identifying which applicants for post-graduate medical education would most benefit from web-based interviews as well as on how residency programs can optimally implement web-based interviews.

### Acknowledgements

The authors acknowledge the statistical analysis assistance of David Juma, MPH, Consulting Coordinator and Senior Consultant, and Briana Wells, MS, Research Analyst, Loma Linda University Research Consulting Group. We also acknowledge the interview coordination assistance of Alex Serafin, MD, Oliver Small, MD, and Leyla Embree, Anesthesiology Residency Program Coordinator, Loma Linda University Medical Center, Loma Linda, CA, USA.

### Conflict of Interest

The authors declare that they have no conflict of interest.
